# Thrombotic microangiopathies: First report of 294 cases from a single institution experience in Argentina

**DOI:** 10.1002/jha2.154

**Published:** 2021-01-19

**Authors:** Célia Dos Santos, Juvenal Paiva, María Lucila Romero, Mara Agazzoni, Ana Catalina Kempfer, Sabrina Rotondo, María Marta Casinelli, María Fabiana Alberto, Analía Sánchez‐Luceros

**Affiliations:** ^1^ Laboratory of Haemostasis and Thrombosis CONICET National Academy of Medicine Institute of Experimental Medicine Buenos Aires Argentina; ^2^ Department of Haemostasis and Thrombosis National Academy of Medicine Haematologic Research Institute “Mariano R. Castex” Buenos Aires Argentina

**Keywords:** ADAMTS13, atypical haemolytic uraemic syndrome, thrombotic microangiopathies, thrombotic thrombocytopenic purpura

## Abstract

**Introduction:**

Introduction: Thrombotic microangiopathies (TMAs) are rare disorders associated with fatal outcomes if left uncared for. However, healthcare problems in developing countries tend to limit medical assistance to patients.

**Methods:**

Methods: We prospectively studied an Argentine cohort of 294 consecutive patients from 2013 to 2016. Patients’ subcategory classification relied on clinical symptoms and presence or absence of trigger events associated with TMA.

**Results:**

Main suspected disorders were the primary TMAs known as thrombotic thrombocytopenic purpura (TTP) (n = 72/294, 24%) and atypical haemolytic uraemic syndrome (aHUS) (n = 94/294, 32%). In acute phase, demographic parameters for acquired TTP (aTTP) (n = 28) and aHUS (n = 47) showed that both groups were characterised by a young median age (37 and 25 years, respectively) and female predominance (60% and 86%). Median of a disintegrin and metalloproteinase with a thrombospondin type 1 motif, member 13 activity was significantly lower in aTTP than in aHUS group (1.4% vs 83%) and was associated with a more severe thrombocytopenia (15 × 10^9^ vs 53 × 10^9^/L). Creatinine (Cr) and urea (Ur) were significantly increased in aHUS compared to aTTP subjects (Cr: 3.7 vs 0.7 mg/dL, Ur: 118 vs 33 mg/dL). Gastrointestinal and neurological symptoms were more frequent in aHUS and aTTP, respectively.

**Conclusion:**

The first description of a TMA cohort in Argentina revealed similar clinical presentations to those of other countries.

## 1 INTRODUCTION

Thrombotic microangiopathies (TMAs) represent a group of rare diseases generally characterised by microangiopathic haemolytic anaemia (MAHA) with thrombocytopenia and organ injury of variable severity [1]. The microvascular lesion includes arterioles and capillary walls thickening, endothelial swelling and detachment, subendothelial accumulation of proteins and cell debris, fibrin and platelet‐rich thrombi obstructing vessel lumina with resultant tissue ischemia [2]. The list of entities belonging to TMA is extensive, and classification remains a challenge while some pathophysiological mechanisms associated with the syndromes are still unclear [3]. Haemolytic uraemic syndrome (HUS) is one of the two main TMA disorders investigated together with thrombotic thrombocytopenic purpura (TTP) in the past few decades. Although the two diseases have overlapping clinical features, HUS and TTP are pathophysiologically distinct entities [4]. TTP is characterised by severe acquired (aTTP) or congenital (cTTP) deficiency of a disintegrin and metalloproteinase with a thrombospondin type 1 motif, member 13 (ADAMTS13). Both forms lead to persistent ultra large von Willebrand factor multimers, platelet activation and microvascular thrombosis [5]. The triad of acute MAHA, thrombocytopenia and acute kidney injury defines HUS. This syndrome is characterised by the typical form, secondary to an infection by Shiga toxin (Stx)‐producing *Escherichia coli* (STEC) and the extremely rare atypical form (aHUS), caused by the dysregulation of the alternative pathway of the complement, leading to its activation. Elucidated pathological mechanisms of aHUS involve genetic or acquired abnormalities identified in complement components or coagulation‐related factors [6]. TMA patient cohorts contributed to depict epidemiological and demographic background of the disease worldwide, except in Latin America where TMA reports are almost nonexistent. The aim of our study was to describe the demographic, laboratory and clinical features of an Argentine cohort of 294 consecutive patients with suspected TMA from 2013 to 2016.

## 2 MATERIAL AND METHODS

### 2.1 Study design and subject population

From January 2013 until December 2016, we prospectively collected plasma samples of 294 consecutive patients, both adults and children, and conducted a retrospective analysis from clinical records. Samples were collected directly from outpatients or shipped to our institution from various health facilities in the country. Demographic (age, sex), clinical (diarrhoea, gastrointestinal and neurological symptoms) and laboratory parameters (haemoglobin, haematocrit, platelets count, lactic acid dehydrogenase‐lactate dehydrogenase [LDH], creatinine, urea) were recorded when available. The study was approved by the Institutional Ethics Committee, and written informed consent was obtained from all subjects.

### 2.2 Definition of syndromes

**FIGURE 1:**
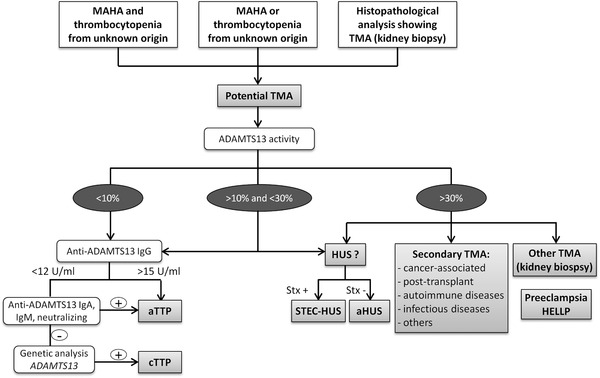
Flow chart of laboratory procedure for patients with clinical suspicion of TMA Abbreviations: ADAMTS13, a disintegrin and metalloproteinase with a thrombospondin type 1 motif, member 13; aHUS, atypical haemolytic uraemic syndrome; aTTP, acquired thrombotic thrombocytopenic purpura; cTTP, congenital thrombotic thrombocytopenic purpura; HELLP, haemolysis, elevated liver enzymes, low platelet count; HUS, haemolytic uraemic syndrome; Ig, immunoglobulins; MAHA, microangiopathic haemolytic anemia; STEC‐HUS, Shiga toxin‐producing *Escherichia coli* HUS; TMA, thrombotic microangiopathy.

Patients who manifested a non‐immune MAHA (haemoglobin < 12 g/dL with schistocytes, elevated LDH), thrombocytopenia (<150x10^9^/L platelets count or a decrease of 25% from baseline) with or without organ injury of variable severity were defined as suspected TMA (Figure 1). Some patients were identified as TMA after performing a renal biopsy. The first step was to measure ADAMTS13 activity. When activity was less than 10%, TTP was confirmed [7]. This diagnostic criterion identifies almost all patients but it was shown that the 10% cutoff level can be problematic in some cases where normal ADAMTS13 activity can be associated with high levels of ADAMTS13 autoantibodies [1]. In the context of our study and to avoid misdiagnosis, we considered a category of patients with an indeterminate range of ADAMTS13 activity between 10% and 30%. Presence of anti‐ADAMTS13 IgG, IgA, IgM or neutralising antibodies confirmed aTTP. In absence of antibodies, the second step was a genetic screening of ADAMTS13 consisting in the amplification of all exons and intron‐exon boundaries by polymerase chain reaction (PCR) followed by Sanger sequencing in order to identify variants causing cTTP. Suspicion of HUS was raised when ADAMTS13 activity was normal in addition to MAHA, thrombocytopenia and kidney failure. In absence of Stx, aHUS was suspected. When a primary event (transplant, cancer, autoimmune diseases and infectious diseases) was associated with TMA symptoms, cases were considered as secondary TMA. Complications during pregnancy like preeclampsia and haemolysis, elevated liver enzymes, low platelet count (HELLP) syndrome were defined as another category of TMA.

### 2.3 Determination of ADAMTS13 parameters in plasma by immunoassay

Measurement of ADAMTS13 activity in human plasma was performed using Technozym ADAMTS13 Activity ELISA (Technoclone, Vienna, Austria). The anti‐ADAMTS13 IgG antibodies assay used Technozym ADAMTS13 Inh ELISA (Technoclone, Vienna, Austria). Patient samples were negative when value was below 12 U/mL, positive when value was above 15 U/mL and considered borderline when value was between 12 and 15 U/mL. Patients with severe deficiency of ADAMTS13 activity but absence of IgG were tested for IgA, IgM or neutralising activity of anti‐ADAMTS13 antibodies (data not shown).

### 2.4 Statistical analyses

Data were processed in GraphPad Prism version 5.01. Descriptive statistics were expressed as medians, ranges and proportions. Comparisons between groups of patients were made using Wilcoxon Mann‐Whitney U test or Fisher's exact test for quantitative or categorical variables, respectively.

## 3 RESULTS

### 3.1 Demographic characteristics and patient distribution in sub‐categories after ADAMTS13 laboratory testing

**FIGURE 2:**
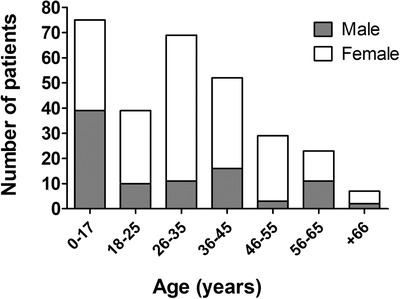
Sex and age distribution of 294 consecutive patients in an Argentine cohort with clinical suspicion of TMA

**FIGURE 3:**
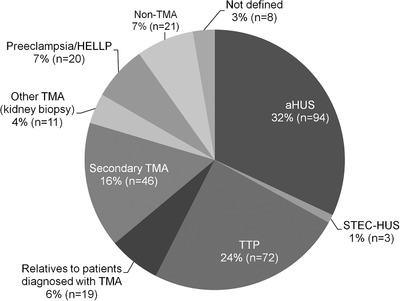
Distribution of the Argentine patient cohort (n = 294) in distinct confirmed or suspected diagnostic categories established through clinical manifestations and determination of ADAMTS13 parameters

Within the 4‐year period (2013‐2016) of our study, 294 patients were referred to our Institution for study of ADAMTS13 parameters. The majority of cases were female patients (n = 202) representing almost 69% of total cohort (Figure 2). Median age of the studied population, including both adults and children, was 31 years (from 0 to 78). Diagnosis of TTP was discarded when normal ADAMTS13 parameters were measured. Patient's clinical manifestations and laboratory were reviewed to classify them in nine sub‐categories (Figure 3). We recorded 72 of 294 (24%) and 94 of 294 (32%) patients with suspected diagnosis of TTP and aHUS, respectively, including relapses and remissions in both groups. Nineteen relatives of probands (6%) were evaluated from those two categories. Only three (1%) STEC‐HUS cases were registered in this cohort. Among secondary TMA patients (n = 46, 16%), a large majority (n = 27, 59%) was related to post‐transplant event (kidney, n = 11; bone‐marrow, n = 3 and liver, n = 1), followed by solid or haematological tumours (n = 12), and seven patients were diagnosed with primary autoimmune diseases, including antiphospholipid syndrome, systemic lupus erythematosus, scleroderma, hepatitis and suspected connective tissue disease. Clinical infectious diseases (HIV, HCV, tuberculosis) were identified in six patients. Six other cases were associated with various events such as pancreatitis (n = 1), uterine fibroids (n = 2), mild pregnancy complication (n = 2) or a case of premature neonate with a significant patent ductus arteriosus. Confirmation of TMA by renal biopsy only, with incomplete pattern of clinical manifestations, was categorised as other TMA (n = 11, 4%). Twenty women (7%), diagnosed with preeclampsia or HELLP syndrome, were studied for ADAMTS13 parameters to discard TTP diagnosis. Finally, eight patients could not be classified due to lack of clinical data from the acute episode or absence of suitable blood samples for ADAMTS13 testing.

### 3.2 ADAMTS13 parameters analysis in TTP versus aHUS patients

**FIGURE 4:**
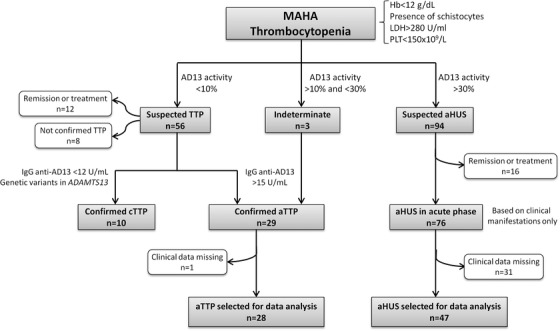
Flow chart of TTP and aHUS patients selected for data analysis Abbreviations: AD13, ADAMTS13; aHUS, atypical haemolytic uraemic syndrome; aTTP, acquired thrombotic thrombocytopenic purpura; cTTP, congenital thrombotic thrombocytopenic purpura; Hb, hemoglobin; Ig, immunoglobulins; LDH, lactate dehydrogenase; MAHA, microangiopathic haemolytic anemia; PLT, platelets; TTP, thrombotic thrombocytopenic purpura.

Data analysis was based on ADAMTS13 activity and clinical characteristics (Figure 4). In presence of MAHA and thrombocytopenia with unknown cause, diagnosis slanted towards suspicion of TTP or aHUS. Diagnosis of TTP was supported in 56 of the 72 patients initially suspected TTP as they presented ADAMTS13 activity of less than 10%. Twelve of those 56 patients were not included in the analysis as they were in remission or receiving treatment during blood testing, as well as eight others for which no antibodies or genetic variants associated with ADAMTS13 were found. Genetic and antibodies testing confirmed cTTP and aTTP diagnosis in 10 and 26 patients, respectively. One patient with aTTP was excluded from the analysis because of incomplete clinical data. We included three patients presenting MAHA and thrombocytopenia in the absence of kidney failure, with an ADAMTS13 activity of between 10% and 30% and positive title of IgG anti‐ADAMTS13 antibodies (Figure 4). Patients with normal ADAMTS13 activity were suspected of aHUS (n = 92). Sixteen were excluded from the analysis because they were referred during remission or treatment, and 31 because of missing clinical data. This selection led to the analysis of 28 and 47 patients with a confirmed laboratory diagnosis of aTTP and with clinical diagnosis of aHUS, respectively.

### 3.3 Demographic and laboratory parameters in TTP versus aHUS patients

**FIGURE 5:**
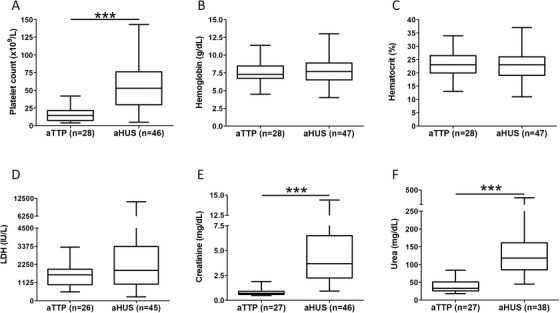
Laboratory parameters analysis in aTTP versus aHUS patients during acute phase ***P < .05, Mann‐Whitney U test. Abbreviations: aHUS, atypical haemolytic uraemic syndrome; aTTP, acquired thrombotic thrombocytopenic purpura; LDH, lactate dehydrogenase.

Median age was higher in aTTP patients compared to cTTP (*P* = .06) and aHUS (*P* < .05) subjects (Table 1). Within the cTTP group, three experienced a first episode during pregnancy, five during childhood and two were diagnosed in adulthood. Acquired TTP and aHUS cohorts were composed of a majority of females whereas sex distribution was similar in cTTP group. Median of ADAMTS13 activity was significantly lower in TTP than in aHUS patients (*P *< .0001). The presence of IgG anti‐ADAMTS13 confirmed aTTP diagnostic (Table 1). Patients with aTTP presented a significant (*P* < .0001) and more severe thrombocytopenia compared with aHUS (Figure 5A). Low haemoglobin and haematocrit levels are characteristics of both TMA syndromes, and no significant difference was observed between aTTP and aHUS (Figures 5B and 5C). While no significant variation in LDH levels was observed between the two groups (Figure 5D), creatinine and urea levels were significantly higher (*P* < .0001) in aHUS than in aTTP (Figures 5E and 5F). Clinical parameters in cTTP patients recorded during first episode were similar to parameters observed in aTTP cohort independently of age at onset (data not shown).

**TABLE 1:** ADAMTS13 parameters of cTTP, aTTP and aHUS patients during acute phase

	cTTP n = 10	aTTP n = 28	aHUS n = 47
Median age in years (minimum‐maximum)	24 (0‐73)	37 (4‐72)	25 (0‐66)
Female (%)	50	86	60
Paediatric population (<18 years old)	5 (50%)	4 (14%)	19 (40%)
ADAMTS13 Activity, median % (minimum‐maximum)	2.4 (0‐10)	1.4 (0‐30)	83 (38‐140)
ADAMTS13 IgG, median U/mL (minimum‐maximum)	4 (1‐8)	69 (23‐860)	3 (0‐19)

Abbreviations: ADAMTS13, a disintegrin and metalloproteinase with a thrombospondin type 1 motif, member 13; aHUS, atypical haemolytic uraemic syndrome; aTTP, acquired thrombotic thrombocytopenic purpura; cTTP, congenital thrombotic thrombocytopenic purpura.

### 3.4 Clinical presentation of extrarenal symptoms and supportive treatments in TTP and aHUS patients

Renal failure is part of aHUS diagnosis but extrarenal manifestations can be commonly observed in TMA disorders. Our cohort showed that aHUS patients were more likely to present gastrointestinal symptoms (40%) than aTTP subjects (4%) (*P* < .0005) (Table 2). Both groups experienced alterations in neurological function; however it was shown to be significantly more frequent in aTTP (57%) than in aHUS (28%) (*P* < .05). Patients suffering from TMA require supportive care earlier. Atypical HUS patients rationally presented a higher rate of dialysis than the aTTP group (62% vs 4%, *P* < .0001). In contrast, corticosteroids seemed to be more frequently administered to patients with aTTP diagnosis (75%) than patients with aHUS clinical manifestations (32%), but this difference was not significant. In the first 48 hours of the acute phase, replacement therapy was applied as frequently to the aTTP cohort as to aHUS individuals (Table 2). The three pregnant women diagnosed with cTTP suffered their first episode of TMA after at least 30 weeks’ gestation and delivered live births following caesarean section. They were all treated with corticoids and plasmapheresis with good response and remained asymptomatic after the crisis. The seven patients who experienced their first episode of cTTP in childhood (n = 5) or adulthood (n = 2) were treated with plasma transfusion and plasmapheresis during the acute phase. Six of them are currently receiving plasma infusion therapy every 15‐20 days.

**TABLE 2:** Clinical manifestations and received treatments in aTTP and aHUS patients during acute phase

	aTTP n = 28 (%)			aHUS n = 47 (%)			***P*‐value** *
	**Presence**	Absence	Unknown	**Presence**	Absence	Unknown	
Gastrointestinal symptoms	**1 (4)**	20 (71)	7 (25)	**19 (40)**	21 (45)	7 (15)	**<.0005**
Neurological symptoms	**16 (57)**	9 (32)	3 (11)	**13 (28)**	23 (49)	11 (23)	**<.05**
Supportive treatments							
Dialysis	**1 (4)**	14 (50)	13 (46)	**29 (62)**	9 (19)	9 (19)	**<.0001**
Corticoids	**21 (75)**	1 (4)	6 (21)	**15 (32)**	1 (2)	31 (66)	**ns**
Transfusion (RBC, plasma or platelets)	**13 (46)**	1 (4)	14 (50)	**26 (55)**	3 (7)	18 (38)	**ns**
Plasmapheresis	**21 (75)**	1 (4)	6 (21)	**26 (55)**	2(4)	19 (41)	**ns**

* Fisher's exact test.

Abbreviations: aHUS, atypical haemolytic uraemic syndrome; aTTP, acquired thrombotic thrombocytopenic purpura; ns, non significant; RBC, red blood cells.

## 4 DISCUSSION

TMAs are rare disorders and life‐threatening entities whose diagnosis is challenging for physicians. Most reports on TMAs have been published mainly in Western Europe [8, 9, 10] and in the United States [11]. More recently, data from the Asian‐Pacific region have started to emerge, including cohort studies from Australia [12], China [13] and Korea [14]. In Latin America, TMA research has mostly focused on STEC‐HUS, described as endemic in Argentina and Uruguay [15]. Our study described the frequency of TMA in an Argentine consecutive cohort for the first time. Compared to a similar study [16], TTP and aHUS were the most frequently suspected entities. A recent retrospective research on a large consecutive cohort of TMA patients showed that less than 10% were TTP, aHUS or STEC‐HUS cases [17]. The authors concluded that divergences in TMA frequency and incidence among studies are likely due to the criteria initially used for patient selection. Compared to studies in the literature, our limited access to complement screening for aHUS diagnosis reduced the scope of our results. However, we were able to confirm TTP diagnosis in 46% of suspected cases in our Argentine cohort. From our results we estimated the annual incidence of aTTP in Argentina to be seven cases, corresponding to 0.2 cases per million people per year. Even if aTTP is more frequent than the inherited form, it is noteworthy to mention that prevalence of cTTP in our Argentine cohort (n = 10/39, 26%) was higher than in the literature. This could be partially explained by the proportion of patients excluded from the analysis (n = 20/59), twelve of whom had been previously diagnosed with aTTP.

Regarding demographic characteristics, both aTTP and aHUS groups were characterised by a predominance of females, as observed previously [18, 19]. Our subject population of suspected TMA was young (median age 31 years), 25% being paediatric patients. In a similar cohort size, median age was slightly higher (34 years) in a Malaysian study (n = 243) with 30% of cases under 24 years [20]. In a German cohort, where the population of TMA paediatric cases was under‐represented (n = 13/232, 6%), the mean age at enrolment was 53 years [21]. Our median age for adult population only was 36 years, which supports literature demonstrating that TMA is more frequent in young adults [1]. The median age difference observed between aTTP and aHUS patients in our cohort reflects that the incidence of aTTP is higher in adults than in children, while aHUS primarily affects children and young adults. We should not discard the possibility that the average lower age of our cohort could be the effect of a referral bias due to inequity in accessing quality healthcare by elderly population in Argentina. This same selection bias, in addition to the difficulties of accessing mid or high‐complexity healthcare centres for TMA diagnosis could have resulted in collecting and evaluating a higher percentage of severe cases compared to other cohorts worldwide.

The commercial chromogenic assay Technozym ADAMTS13 Activity ELISA (Chr‐VWF73) is one of the most popular clinical laboratory methods for ADAMTS13 activity. However lack of standardisation in methodologies and sources of discrepancy between them can be challenging if carried out by non‐expert laboratories. Our reference centre has accumulated experience of studying TTP patients over the past 20 years [22, 23]. This expertise is important in the management of cases that might present divergence between laboratory results and clinical manifestations, as observed by George [24]. Samples of patients that present overlapping results and show inconsistency with clinical manifestations are rare but may occur [25, 26]. A recent consensus on clinical utility of assays recommends repeating ADAMTS13 testing in TMA patients with an indeterminate range of 10‐20% of activity [27]. In our cohort, 10% (n = 3) of our confirmed aTTP patients presented a normal ADAMTS13 activity (between 10% and 30%). Repeating the assay using a new sample from the patients was challenging as subjects would usually receive replacement therapy at the earliest. Additional methods in the laboratory were then useful to support a diagnosis including evaluation of ultra large VWF multimers and collagen binding assay. For aHUS diagnosis, no complement biomarker alone has proved its utility, but various serological laboratory tests are of interest if examined altogether [28]. However, the lack of resources encountered by developing countries is limiting their capacity to manage rare syndromes like TMA properly, as exposed by a recent publication from India [29]. Lack of clinical or laboratory data can be a limiting factor when studying TMA. In the past few years, the PLASMIC score was validated in the literature as a useful tool to predict TTP early [30, 31, 32]. This prognostic score as well as others was developed with the objective of reducing the time from diagnosis to treatment [33]. The use of a prognostic score is of great interest in Argentina as resources for TMA diagnosis can be limited. Moreover, our results clearly showed that the two parameters described as useful and reliable to predict severe ADAMTS13 deficiency, platelet count and serum creatinine [34], were indeed both statistically significant when comparing patients from TTP and aHUS groups.

Not only is therapeutic approach of TMA still challenging due to the lack of treatments, but also because of diagnostic uncertainty. Our turnaround time to measure ADAMTS13 activity can be problematic and cause delays in considering other diagnoses and/or treatments, which can impact patient outcomes. In that context, patients with suspected TMA might receive plasma exchange regardless of their real need. That being said, recommendations for TMA treatment are to initiate plasmapheresis as soon as possible [35]. Consequently, our cohort is composed of a high rate of aHUS patients treated with plasmapheresis (Table 2) who also received supportive care including dialysis and blood component transfusions. Meanwhile, initial plasma exchange in most of aTTP patients was supplemented with corticosteroids administration. Additionally, a B‐cell depletion molecule, rituximab, is frequently used in immune‐mediated TTP as an adjunction to initial therapy or as first‐line treatment [36]. Nevertheless, poor capacity to follow‐up patients prevented us from including the number of cases treated with rituximab in our cohort. Eculizumab for aHUS therapy [37] is not authorised by the National Drugs, Food and Medical Technology Administration (ANMAT) in Argentina and can only be accessed for compassionate use. Combined with its high cost, delays in the administration of the drug are common and may affect patient recovery. In developing countries, improving the diagnosis timeframe by means of early testing will be more relevant if access to new and expensive treatments is increased, such as caplacizumab, approved by FDA in 2019 for TTP [38].

In conclusion, our study of 294 suspected Argentine TMA patients represents, to the best of our knowledge, the largest cohort ever reported in Latin America. From this dataset, clinical manifestations and determination of ADAMTS13 parameters allowed us to describe a high frequency of primary TMA corresponding to TTP and aHUS cases. This study provides the first report of epidemiological information about these rare diseases in Argentina. Accumulating data and knowledge about all TMA disorders aims to offer healthcare professionals the possibility to improve management, treatment and prevention of affected patients.

## CONFLICT OF INTEREST

Célia Dos Santos and Analía Sánchez‐Luceros have received speaker honoraria from Raffo. Analía Sánchez‐Luceros has received educational grants (2015‐2017) from Alexion‐Argentina and grant for clinical support from Raffo (since 2018). Juvenal Paiva, María Lucila Romero, Mara Agazzoni, Ana Catalina Kempfer, Sabrina Rotondo, María Marta Casinelli and María Fabiana Alberto have no conflict of interests.

## AUTHOR CONTRIBUTIONS

Célia Dos Santos designed the study, collected and analysed data and wrote the manuscript. Célia Dos Santos, Juvenal Paiva, Ana Catalina Kempfer, Sabrina Rotondo, María Marta Casinelli and María Fabiana Alberto performed the laboratory studies. Mara Agazzoni collected data and provided clinical support. María Lucila Romero and Analía Sánchez‐Luceros designed the study, contributed to patient recruitment and provided clinical support. María Lucila Romero, María Fabiana Alberto and Analía Sánchez‐Luceros revised the manuscript. All authors approved the final version for journal submission.

## Data Availability

The data that support the findings of this study are available on request from the corresponding author. The data are not publicly available due to privacy or ethical restrictions.
